# Supplementary material for: Comparative multiomics analysis of cell physiological state after culture in a basket bioreactor
Source: Sci Rep. 2022 Nov 23;12:20161. doi: 10.1038/s41598-022-24687-4 (PMC9686226; doi:10.1038/s41598-022-24687-4)
Supplement: Supplementary file 1 — Supplementary Information 1. [file 41598_2022_24687_MOESM1_ESM.zip › raw data/Transcriptomic raw data/6.Enrichment/1.GO/TE1vsTC1/GSEA/TE1/GROWTH/GROWTH(GO_0040007).html]

Details for gene set GROWTH(GO:0040007)[GSEA]


|  || Dataset | readcount.sample |
| Phenotype | sample.cls |
| Upregulated in class | TE1 |
| GeneSet | GROWTH(GO:0040007) |
| Enrichment Score (ES) | 0.5815503 |
| Normalized Enrichment Score (NES) | 1.7594355 |
| Nominal p-value | 0.0 |
| FDR q-value | 0.20460911 |
| FWER p-Value | 0.494 |
Table: GSEA Results Summary

  

Fig 1: Enrichment plot: GROWTH(GO:0040007)
  
Profile of the Running ES Score & Positions of GeneSet Members on the Rank Ordered List

  

| PROBE | DESCRIPTION   (from dataset) | GENE SYMBOL | GENE\_TITLE | RANK IN GENE LIST | RANK METRIC SCORE | RUNNING ES | CORE ENRICHMENT || 1 | 103243987 | BMP5 |  |  | 605 | 18.075 | 0.2593 | Yes |
| 2 | 103246725 | BMP2 |  |  | 805 | 5.136 | 0.3318 | Yes |
| 3 | 103246429 | HTRA3 |  |  | 853 | 4.658 | 0.4035 | Yes |
| 4 | 103230001 | LOC103230001 |  |  | 876 | 4.440 | 0.4729 | Yes |
| 5 | 103224516 | CCN1 |  |  | 1325 | 2.745 | 0.4963 | Yes |
| 6 | 103220708 | GDF7 |  |  | 1668 | 2.296 | 0.5174 | Yes |
| 7 | 103240368 | CCN2 |  |  | 2247 | 1.961 | 0.5226 | Yes |
| 8 | 103220492 | CRIM1 |  |  | 2308 | 1.931 | 0.5506 | Yes |
| 9 | 103227748 | MUSTN1 |  |  | 2360 | 1.908 | 0.5785 | Yes |
| 10 | 103237164 | GDF6 |  |  | 2906 | 1.731 | 0.5816 | Yes |
| 11 | 103226169 | INHBA |  |  | 3790 | 1.558 | 0.5667 | No |
| 12 | 103235099 | CNTF |  |  | 6032 | 1.299 | 0.4871 | No |
| 13 | 103238487 | GDF11 |  |  | 7904 | 1.150 | 0.4217 | No |
| 14 | 103235872 | BMP3 |  |  | 9252 | 1.069 | 0.3784 | No |
| 15 | 103226113 | IGFBP3 |  |  | 9479 | 1.055 | 0.3850 | No |
| 16 | 103235635 | IGFBP7 |  |  | 9972 | 1.022 | 0.3792 | No |
| 17 | 103230082 | TGFB2 |  |  | 10295 | 1.004 | 0.3807 | No |
| 18 | 103240560 | CCN6 |  |  | 10893 | 0.963 | 0.3693 | No |
| 19 | 103237344 | CCN3 |  |  | 11356 | 0.935 | 0.3634 | No |
| 20 | 103234729 | TGFB1 |  |  | 12493 | 0.863 | 0.3263 | No |
| 21 | 103217825 | IGFBP2 |  |  | 16478 | 0.607 | 0.1578 | No |
| 22 | 103222207 | BMP6 |  |  | 16936 | 0.574 | 0.1465 | No |
| 23 | 103243784 | BMP7 |  |  | 17287 | 0.550 | 0.1395 | No |
| 24 | 103219167 | IGFBPL1 |  |  | 17456 | 0.535 | 0.1405 | No |
| 25 | 103229007 | BMP4 |  |  | 17488 | 0.533 | 0.1475 | No |
| 26 | 103229367 | TGFB3 |  |  | 17697 | 0.516 | 0.1464 | No |
| 27 | 103216638 | HTRA1 |  |  | 18068 | 0.484 | 0.1375 | No |
| 28 | 103219765 | NINJ1 |  |  | 18134 | 0.477 | 0.1422 | No |
| 29 | 103217826 | IGFBP5 |  |  | 18397 | 0.451 | 0.1376 | No |
| 30 | 103225060 | BMP8B |  |  | 18737 | 0.417 | 0.1291 | No |
| 31 | 103216405 | KAZALD1 |  |  | 19057 | 0.385 | 0.1209 | No |
| 32 | 103229999 | LOC103229999 |  |  | 19125 | 0.378 | 0.1239 | No |
| 33 | 103216911 | INHBB |  |  | 19298 | 0.362 | 0.1219 | No |
| 34 | 103243511 | IGFBP4 |  |  | 19698 | 0.316 | 0.1091 | No |
| 35 | 103225049 | BMP8A |  |  | 19704 | 0.316 | 0.1139 | No |
| 36 | 103245540 | CCN5 |  |  | 19971 | 0.278 | 0.1064 | No |
| 37 | 103237471 | CCN4 |  |  | 20747 | 0.139 | 0.0740 | No |
| 38 | 103221452 | ESM1 |  |  | 21112 | 0.000 | 0.0577 | No |
Table: GSEA details
[plain text format]

  

Fig 2: GROWTH(GO:0040007)
  
Blue-Pink O' Gram in the Space of the Analyzed GeneSet

  

Fig 3: GROWTH(GO:0040007): Random ES distribution
  
Gene set null distribution of ES for
 **GROWTH(GO:0040007)**
